# Responding to COVID-19 vaccine-related safety events: WHO Western Pacific regional experience and lessons learned

**DOI:** 10.5365/wpsar.2023.14.2.1016

**Published:** 2023-06-24

**Authors:** Heeyoun Cho, Ananda Amarasinghe, Yoshihiro Takashima

**Affiliations:** a Vaccine-Preventable Diseases and Immunization, Division of Programs for Disease Control, World Health Organization Regional Office for the Western Pacific, Manila, Philippines.

## Abstract

**Problem:**

Novel vaccines were developed in an unprecedentedly short time in response to the global coronavirus disease (COVID-19) pandemic, which triggered concerns about the safety profiles of the new vaccines. This paper describes the actions and outcomes of three major adverse events of special interest (AESIs) reported in the World Health Organization’s (WHO’s) Western Pacific Region: anaphylaxis, thrombosis with thrombocytopenia syndrome (TTS) and post-vaccination death.

**Context:**

During the large-scale introduction of various novel COVID-19 vaccines, robust monitoring of and response to COVID-19 vaccine safety events were critical.

**Action:**

We developed and disseminated information sheets about anaphylaxis and TTS; provided tailor-made training for anaphylaxis monitoring and response, webinars about TTS and AESIs, and an algorithm to support decision-making about AESIs following immunization; as well as provided country-specific technical support for causality assessments, including for possible vaccination-related deaths.

**Outcome:**

Each major vaccine event and situation of high concern was responded to appropriately and in a timely manner with comprehensive technical support from WHO. Our support activities have not only strengthened countries’ capacities for vaccine safety surveillance and response, but also enabled countries to decrease the negative impact of these events on their immunization programmes and maintain the confidence of health-care professionals and the general population through proactive delivery of risk communications.

**Discussion:**

This paper summarizes selected, major AESIs following COVID-19 vaccination and responses made by WHO’s Regional Office for the Western Pacific to support countries. The examples of responses to vaccine safety events during the pandemic and unprecedented mass vaccination campaigns could be useful for countries to adopt, where applicable, to enhance their preparation for activities related to monitoring vaccine safety.

## PROBLEM

The World Health Organization (WHO) declared a global coronavirus disease (COVID-19) pandemic in March 2020. (*1*) Novel COVID-19 vaccines were developed in an unprecedentedly short time, with WHO listing the first COVID-19 vaccine, the Comirnaty (Pfizer-BioNTech) COVID-19 mRNA vaccine, for emergency use in December 2020. (*2*) This was followed by other COVID-19 vaccines that used various platforms, including an adenovirus vector–based vaccine, an inactivated vaccine and a protein subunit vaccine.

Large-scale vaccination campaigns were conducted globally, which triggered concerns about the safety profile of the vaccines, particularly about rare serious adverse events of special interest (AESIs). AESIs, a subset of serious adverse events following immunization (AEFIs), are defined as preidentified and predefined events that are medically significant and have the potential to be causally associated with a vaccine product and that need to be carefully monitored and confirmed or discounted by further specific studies. AESIs require careful monitoring – ideally through an active surveillance system – to determine whether the event is truly associated with a vaccine or vaccination. (*3*)

This paper describes the actions taken by WHO’s Regional Office for the Western Pacific and the outcomes associated with three major high-impact AESIs reported in the Region, as well as public and programme managers’ concerns about them: anaphylaxis, thrombosis with thrombocytopenia syndrome (TTS) and post-vaccination death.

## CONTEXT

Various novel COVID-19 vaccines have been introduced globally since late 2020 as part of the public health emergency response to the pandemic. Before the COVID-19 vaccine roll out, countries in WHO’s Western Pacific Region started preparing for COVID-19 vaccine safety surveillance. COVID-19 vaccination has been the largest mass vaccination programme in immunization history, covering wide age groups across all geographical regions. The delivery of millions of COVID-19 vaccine doses within less than 2 years led to a large number of reported serious AEFIs and AESIs.

### Anaphylaxis

Anaphylaxis is a rare but serious allergic reaction that is occasionally fatal, if not treated quickly and properly. (*4*) It is a well known serious AEFI of many vaccines used for routine immunization, including the hepatitis B vaccine, human papillomavirus vaccine and measles-containing vaccines. The expected anaphylaxis rate of these non-COVID-19 vaccines is approximately 1–6 per 1 million doses. (*4*) During the early stage of the COVID-19 vaccination roll out, there was concern about the relatively high reporting rates of anaphylaxis observed globally and in the Western Pacific Region. For example, 21 cases of anaphylaxis were reported following administration of approximately 1.9 million doses of the Pfizer-BioNTech COVID-19 vaccine in the United States of America (11.1/1 million doses) during 2 weeks in December 2020. (*5*) Based on internal data from the Regional Office for the Western Pacific from four countries’ weekly AEFI reports, as of April 2021, the reporting rate for anaphylaxis ranged from approximately 3.2 to 127.9 per 1 million doses for four different COVID-19 vaccines, including those by Vaxzevria (AstraZeneca), Pfizer-BioNTech, CoronaVac (Sinovac) and BBIBP-CorV (Sinopharm).

The high number of anaphylaxis diagnoses may be largely due to increased awareness of anaphylaxis and a high index of clinical suspicion among health-care workers. Overdiagnosis of anaphylaxis is not uncommon and has been reported for both COVID-19 vaccines and routine immunizations. (*6*, *7*) Some countries in the Western Pacific Region have suboptimal capacity, particularly at the subnational level, for emergency responses and management of anaphylaxis following immunization. Both overdiagnosis and underdiagnosis of anaphylaxis are concerns. Overdiagnosis is safer than underdiagnosis, which can lead to a potentially fatal outcome due to a delay in providing the proper treatment. However, overdiagnosis can negatively impact a vaccination programme and result in declining vaccine acceptance. The overuse of adrenaline for treating suspected anaphylaxis is another concern, which can also cause adverse health outcomes. (*8*)

### Thrombosis with thrombocytopenia syndrome

TTS was one of the earliest AESIs reported during the post-authorization phase of COVID-19 vaccines. As of 31 August 2021, TTS reporting rates ranged from 0.2 in Asian countries to 17.6 in Nordic countries per  1 million doses. (*9*) This newly reported rare AESI following administration of COVID-19 adenovirus vector–based vaccines (e.g. AstraZeneca and Ad26.COV 2-S [Johnson & Johnson] vaccines) has raised great concern not only within the Western Pacific Region but also globally because TTS can be fatal and has many unknown characteristics in the context of novel COVID-19 vaccines. Particularly during the early stage of the COVID-19 vaccination roll out, in many low- and middle-income countries with limited capacity for diagnosing and assessing potential TTS cases, detection and reporting were challenging, primarily due to the uncertainty of pathogenesis, the complicated clinical and laboratory presentations, and the lack of a clear case definition. Potential TTS cases might not be detected and reported in resource-limited settings, considering there is a significant gap in diagnostic capacity between high-income countries and low- and middle-income countries.

### Post-vaccination deaths

The WHO Strategic Advisory Group of Experts on immunization has recommended that elderly people and people with comorbidities should be among the highest-priority groups for COVID-19 vaccination to minimize disease severity and mortality. (*10*) Considering the high risk of mortality among these groups following any medical condition, it would be anticipated that deaths in these groups following COVID-19 vaccination could be falsely attributed to the vaccine or vaccination. This highlights the importance of using caution when interpreting reporting rates of deaths following immunization as well as the importance of conducting thorough investigations followed by comprehensive causality assessments for all post-vaccination deaths. The availability of background mortality rates, particularly cause-specific rates, is important and necessary to ensure a valid population-based causality assessment can be conducted at the country level.

## ACTIONS

### Anaphylaxis

Timely diagnosis and management are critical to avoid fatal anaphylaxis following COVID-19 vaccination. Therefore, we focused on increasing awareness of and facilitating preparedness for managing anaphylaxis, even in limited-resource settings. We developed and distributed an anaphylaxis information sheet tailored to the COVID-19 vaccination response to country focal points for COVID-19 vaccination and to WHO Country Office teams; the information sheet included the case definition, clinical features, expected rates after vaccination and information about basic initial treatment. We also periodically shared updated anaphylaxis rates and trends to help inform the safety profiles of the COVID-19 vaccines.

In addition, to enhance country-specific capacity for anaphylaxis response and management, we provided online refresher training that focused on proper diagnosis and appropriate and timely clinical management, although anaphylaxis is not a new AEFI. During November–December 2021, clusters of anaphylaxis cases following administration of various COVID-19 vaccines were reported from multiple provinces in Viet Nam. Investigations revealed there was a likelihood of overdiagnosis of anaphylaxis. In response to these reported clusters of cases, we facilitated a comprehensive training course in December 2021 for clinicians at the national and provincial levels, conducted by the Ministry of Health, about managing anaphylaxis, with particular focus on differential diagnosis and the rational use of adrenaline.

### Thrombosis with thrombocytopenia syndrome

We provided an information sheet on TTS, similar to the one for anaphylaxis, which included a technical guide to diagnosis and management. In addition, with the support of the National Centre for Immunization Research and Surveillance in Australia, we held a webinar on AESIs related to COVID-19 vaccination, including TTS, to provide the most updated information to enable health-care workers to detect and report potential cases early. Particularly for Pacific island countries and areas, where clinical specialists and diagnostic tools are limited, we provided joint virtual trainings and telemedicine consultations for clinical assessment of individual AESI cases in collaboration with WHO country offices and relevant external partners, including the National Centre for Immunization Research and Surveillance.

**Fig. 1:**
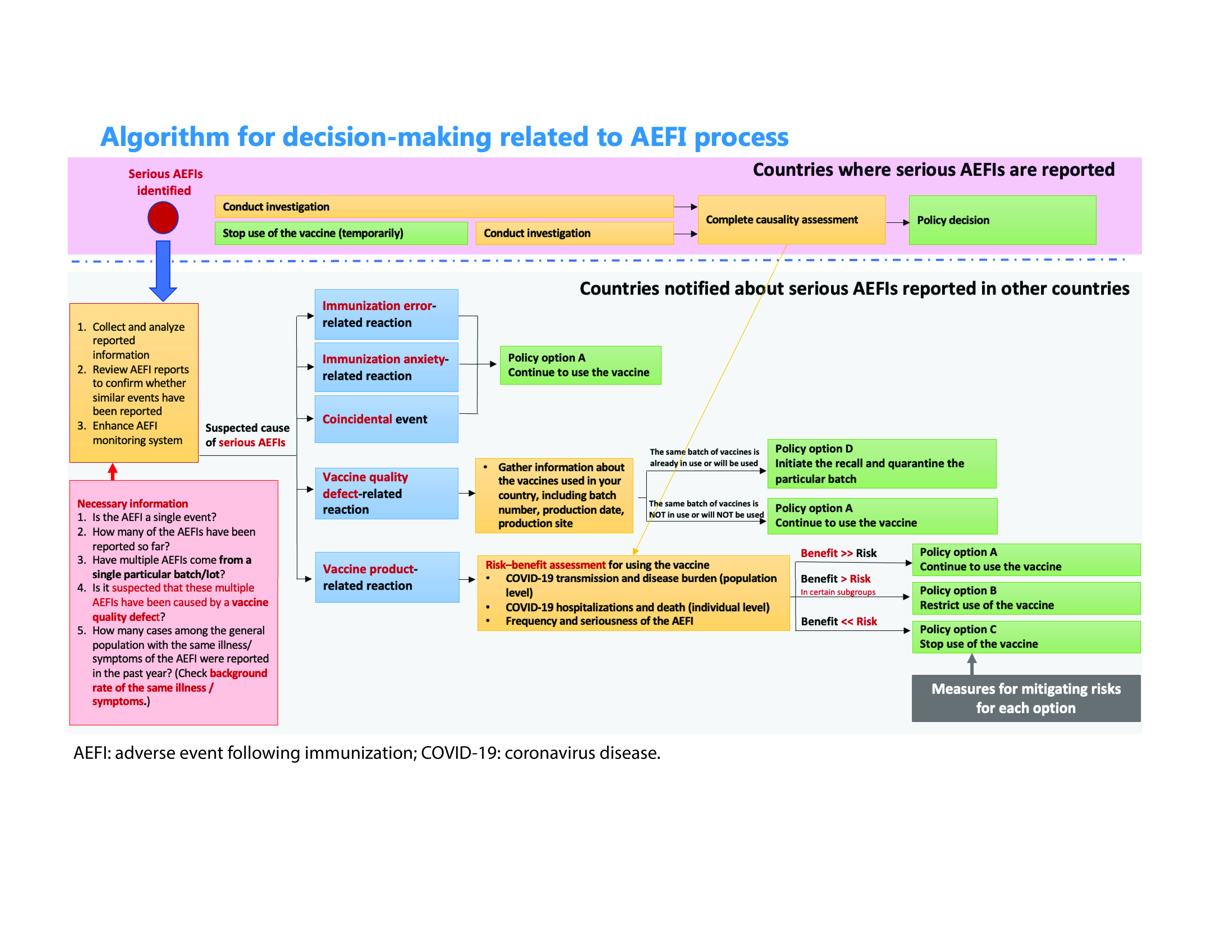
Algorithm for decision-making related to AEFIs following COVID-19 immunization, WHO Western Pacific Region, July 2022

We also developed an algorithm to support the decision-making process at the country level for when a rare but serious AESI was reported. The simplified algorithm (**Fig. 1**) displayed possible policy options (A–D) for countries that were vigilant about AESIs reported in other countries although not necessarily detected in their own. These options were primarily based on a risk–benefit assessment. For example, policy option A, which is to continue using the vaccine with risk mitigation measures, describes a situation in which the benefits of continued vaccination outweigh a potential risk even if there is a possible association between a vaccine and an AESI. This proactive development of the algorithm enabled countries to continue COVID-19 vaccination without unnecessary suspension of the use of a given vaccine.

[**insert** Figure 1]

### Post-vaccination deaths

We have provided ongoing technical assistance to investigations and causality assessments of AESIs and deaths since the COVID-19 vaccination roll out in 2021. This was done through workshops and consultations for members of national AEFI committees in countries including Brunei Darussalam, the Lao People's Democratic Republic, Malaysia, the Philippines and Pacific island countries and areas.

We conducted an in-depth analysis of a subset of post-vaccination deaths reported in the Philippines from March to May 2021 to further support the assessments of the national AEFI committee looking into deaths possibly related to a specific batch of vaccines.

## OUTCOMES

### Anaphylaxis

The tailor-made tools disseminated in a timely manner to countries triggered staff awareness and have contributed to better preparedness for detecting and managing anaphylaxis. Intensive awareness of possibly high observed reporting rates has led to more confidence among immunization staff and clinicians in being cautious in interpreting and responding to them. Based on the authors’ observations and continuous communication with WHO country offices, the periodic sharing of monitoring and updates of anaphylaxis rates and trends appears to have significantly contributed to avoiding unwarranted concerns from national stakeholders.

By March 2022, anaphylaxis reporting rates following the administration of various COVID-19 vaccines in countries in the Western Pacific Region had gradually declined to 0.3–13.7 cases per 1 million doses. Despite the very high reporting rates for anaphylaxis observed early on during COVID-19 vaccination, the more stable rates reported by March 2022 offer reassurance that they are comparable to those of many other vaccines used globally in immunization programmes. (*3*) This is an important observation and a lesson learned: during the period when any new vaccine is introduced, there is a possibility of higher-than-expected reaction rates or rates that are even higher than the background rates for AESIs. However, over time the rates will return to the expected range as a result of the high number of doses being administered (i.e. with a larger denominator) for any given vaccine. Thus, caution should be used when interpreting and responding to the observed rates of serious AEFIs or AESIs during the early stage of a vaccine roll out.

Additionally, during November–December 2021, clusters of anaphylaxis cases were reported after administration of various COVID-19 vaccines in multiple provinces in Viet Nam. The WHO-supported investigations revealed the likelihood of overdiagnosis of anaphylaxis. The situation was improved promptly and rectified by providing comprehensive training for clinicians at the national and provincial levels.

### Thrombosis with thrombocytopenia syndrome

Our tools were extensively used to update the knowledge of health-care workers, COVID-19 vaccination focal points and policy-makers, all of whom needed specific information about the diagnosis, clinical management and safety profile of this new AESI identified after authorization of the vaccines. Collaborative telemedicine consultations provided real-time support to clinicians, who could be reassured of their ability to clinically manage this complex adverse event and avoid or minimize any potential serious consequences.

### Post-vaccination deaths

After providing technical assistance to the Philippines, we conducted an analysis of a subset of deaths reported there following COVID-19 vaccination occurring from March to May 2021, and we were able to support the conclusion of the national AEFI committee that cause-specific death rates following COVID-19 vaccination were significantly lower than the background rates in the Philippines. This analysis reassured stakeholders by ruling out a possible safety signal for a certain batch of vaccines. Further, after causality assessments, these deaths were determined not to be causally associated with the vaccines.

These country-support activities have not only strengthened countries’ capacities for causality assessment, but also enabled them to decrease the negative impact of these events on their immunization programmes and maintain the confidence of health-care professionals and the general population by delivering proactive risk communications.

## Discussion

This paper summarized a few major adverse events that occurred following COVID-19 vaccination and the responses by WHO’s Regional Office for the Western Pacific to support countries when specific vaccine safety events occurred. Lessons learned from these experiences were (i) the importance of ensuring correct interpretation of observed AESI rates over time (e.g. anaphylaxis) when a new vaccine is being introduced; (ii) the importance of being prepared to provide appropriate management of and responses to newly reported AESIs (e.g. TTS) in a timely manner; and (iii) a need to implement evidence-based decision-making following serious AEFIs, AESIs and post-vaccination deaths after thorough and scientific investigation and causality assessment to sustain the public’s trust in vaccination. However, this paper has shared only limited quantitative data and instead has focused primarily on sharing the lessons learned, which it is hoped will benefit future preparedness activities for manging safety events when new vaccines are introduced.

The examples presented in this paper about COVID-19 vaccine safety events and responses during the pandemic and the associated unprecedented mass vaccination campaigns could be useful for countries seeking to strengthen their surveillance of and response to events possibly related to vaccine safety. Countries’ capacities and preparedness for vaccine and immunization safety monitoring and responses are important to ensure continuing large-scale introduction of new vaccines. Moreover, if COVID-19 vaccination is to continue as part of a life-course approach – that is, to be integrated with regular immunization programmes – these responses will be useful guiding examples to aid in planning and implementing effective risk communication strategies to prevent vaccine hesitancy, particularly pertaining to vaccine safety concerns, and maintain trust in and demand for regular immunization.

## References

[1] WHO Director-General’s opening remarks at the media briefing on COVID-19 – 11 March. Geneva: World Health Organization; 2020. Available from: https://www.who.int/director-general/speeches/detail/who-director-general-s-opening-remarks-at-the-media-briefing-on-covid-19 — 11-march-2020, accessed 9 March 2023.

[2] WHO issues its first emergency use validation for a COVID-19 vaccine and emphasizes need for equitable global access. Geneva: World Health Organization; 2020. Available from: https://www.who.int/news/item/31-12-2020-who-issues-its-first-emergency-use-validation-for-a-covid-19-vaccine-and-emphasizes-need-for-equitable-global-access, accessed 9 March 2023.

[3] COVID-19 vaccines: safety surveillance manual. Geneva: World Health Organization; 2020. Available from: https://apps.who.int/iris/handle/10665/338400, accessed 9 March 2023.

[4] Immunization safety surveillance: guidelines for immunization programme managers on surveillance of adverse events following immunization, third edition. Manila: WHO Regional Office for the Western Pacific; 2016. Available from: https://apps.who.int/iris/handle/10665/208262, accessed 9 March 2023.

[5] CDC COVID-19 Response TeamFood and Drug Administration. Allergic reactions including anaphylaxis after receipt of the first dose of Pfizer-BioNTech COVID-19 vaccine – United States, December 14–23, 2020. MMWR Morb Mortal Wkly Rep. 2021 Jan 15;70(2):46–51. 10.15585/mmwr.mm7002e1 33444297 PMC7808711

[6] Carr BZ, Spriggs K, Ojaimi S, Leahy E, Barnes SL. Re-assessing reactions to influenza vaccination initially classified as vaccine allergies. Med J Aust. 2022 Aug 1;217(3):155–6. 10.5694/mja2.51593 35656786

[7] Greenhawt M, Abrams EM, Oppenheimer J, Vander Leek TK, Mack DP, Singer AG, et al. The COVID-19 pandemic in 2021: avoiding overdiagnosis of anaphylaxis risk while safely vaccinating the world. J Allergy Clin Immunol Pract. 2021 Apr;9(4):1438–41. 10.1016/j.jaip.2021.01.022 33529722 PMC7847187

[8] Brief overview of anaphylaxis as an adverse event following immunization (AEFI) and practical guidance on its identification, case management and response in a primary care setting. Geneva: World Health Organization; 2021. Available from: https://apps.who.int/iris/handle/10665/342195, accessed 9 March 2023.

[9] Soboleva K, Shankar NK, Yadavalli M, Ferreira C, Foskett N, Putsepp K, et al. Geographical distribution of TTS cases following AZD1222 (ChAdOx1 nCoV-19) vaccination. Lancet Glob Health. 2022 Jan;10(1):e33–4. 10.1016/S2214-109X(21)00545-3 34919849 PMC8670752

[10] WHO SAGE roadmap for prioritizing uses of COVID-19 vaccines: an approach to optimize the global impact of COVID-19 vaccines, based on public health goals, global and national equity, and vaccine access and coverage scenarios, first issued 20 October 2020, updated: 13 November 2020, updated: 16 July 2021, latest update: 21 January 2022. Geneva: World Health Organization; 2022. Available from: https://apps.who.int/iris/handle/10665/351138, accessed 9 March 2023.

